# Inflammation and oxidative stress, rather than hypoxia, are predominant factors promoting angiogenesis in the initial phases of atherosclerosis

**DOI:** 10.3892/mmr.2015.3800

**Published:** 2015-05-19

**Authors:** WEIGANG XIAO, ZHENHUA JIA, QIUYAN ZHANG, CONG WEI, HONGTAO WANG, YILING WU

**Affiliations:** 1 Graduate School Hebei Medical University, Shijiazhuang, Hebei 050017, P.R. China; 2 Cardiovascular Medicine, Affiliated Yiling Hospital of Hebei Medical University, Shijiazhuang, Hebei 050091, P.R. China; 3 Pharmacology Lab, Yiling Pharmaceutical Research Institute, Shijiazhuang, Hebei 050035, P.R. China; 4 Key Laboratory of State Administration of Traditional Chinese Medicine, Shijiazhuang, Hebei 050035, P.R. China; 5 Hebei Key Laboratory of Collateral Disease, Shijiazhuang, Hebei 050035, P.R. China

**Keywords:** atherosclerosis, angiogenesis, inflammation, hypoxia, oxidative stress

## Abstract

Micro-angiogenesis in the arterial wall has been observed during the development and progression of atherosclerosis. The aim of the present study was to examine whether inflammation, oxidative stress and hypoxia are involved in the process of early atherosclerotic micro-angiogenesis. A total of 24 rabbits were randomly divided into a normal diet group or a high-cholesterol (HC) diet group and were fed the corresponding diets for 4 weeks. The microvessel density (MVD), level of hypoxia and the levels of inflammatory markers and antioxidants in the arterial wall were detected using immunohistochemical and molecular biological techniques, respectively. The present results demonstrated that the MVD in the HC group was significantly higher (P<0.01) than that observed in the rabbits, which were provided with a normal diet, while hypoxia-inducible factor-1α levels did not exhibit marked changes in either of the two groups (P>0.05). The levels of inflammatory markers and antioxidants were significantly different between the two groups (P<0.05). The present study demonstrated that the primary factors, which promote micro-angiogenesis are possibly associated with an increase in inflammation and a decrease in the levels of antioxidants, as tissue hypoxia in the arterial wall at this stage was not evident.

## Introduction

Atherosclerosis is a chronic inflammatory pathological process, involving multiple factors and often occurring in large and medium-sized arteries (1). Atherosclerosis involves lipid deposition, inflammation (2), oxidative stress (3), angio-genesis (4) and other pathological processes and is associated with hypertension, hyperglycaemia, aging and various other risk factors (5). Previous studies have revealed that microvessels of the vessel walls, namely, the vasa vasorum (VV) have an important role in maintaining the homeostasis of the vessel wall and their distribution density is closely associated with a susceptibility to atherosclerosis. Therefore, the VV density is relatively higher in areas that are prone to atherosclerosis. Notably, the VV density of the coronary arteries is significantly higher than that of the renal arteries and iliac arteries in humans (6), while, in pigs, the VV densities of the coronary artery, renal artery, carotid artery and femoral artery are decreased (7). In addition, the distribution of the VV is associated with atherosclerotic lesion formation and distribution among the different vascular beds in apolipoprotein E^−/−^/low density lipoprotein (LDL)^−/−^ double knockout mice (8).

The adventitial VV is a major source of advanced atherosclerotic plaque angiogenesis, which accounts for ~96.6% of the total number of angiogeneses within plaques (9). This angiogenesis is one of the important indicators of vulnerable plaques. The inhibition of plaque angiogenesis is able to effectively stabilize vulnerable plaques, decrease the risk of plaque rupture, and reduce macrophage accumulation and the progression of advanced atherosclerosis (10). A previous study revealed that coronary VV angiogenesis occurred during the hyperlipidemia period and prior to the development of endothelial dysfunction, a hallmark of the early atherosclerotic lesion (11). The aforementioned studies demonstrated that VV angiogenesis occurs throughout the development of the atherosclerotic plaque and the progression process, causing negative effects. A previous study hypothesised that the VV angiogenesis of early atherosclerosis improved the hypoxic condition of the arterial walls and occurred as a result of hypoxia alone (12). However, there are also studies indicating that angiogenesis in the early stages of the formation of atherosclerotic lesions in human carotid arteries potentially contributes to plaque development (13). In addition, hypercholesterolemia does not alter the delivery of oxygen to the artery wall prior to the formation of atherosclerotic lesions (14). Therefore, the arterial wall does not exhibit marked levels of hypoxia during the period of hypercholesterolemia. With regards to the discrepancies over its beneficial or detrimental effects and whether it involves hypoxia or not, the role of angiogenesis in early atherosclerosis requires further elucidation.

Intimal thickening is one of the causes of the arterial wall oxygen deficit and hypoxia is able to also cause advanced atherosclerotic plaque angiogenesis. However, VV angiogenesis has been observed to appear during the initial stages of atherosclerosis, which are induced by hyperlipidemia (11). Whether angiogenesis at this time is associated with hypoxia and whether the inflammation or oxidative stress level is involved in this form of angiogenesis remains to be elucidated. Previous studies have focussed on angiogenesis in the atherosclerotic plaque and the effect of drug treatments on these angiogeneses, while the angiogenic mechanism in the initial stage of atherosclerosis has not been fully examined.

The present study aimed to assess whether inflammation, oxidative stress and hypoxia were all involved in the process of angiogenesis of early atherosclerotic lesions.

## Materials and methods

### Animals

The disposal and management for all animals complied with the rules for animal management of the Ministry of Science and Technology of the People's Republic of China (documentation 398, 2006) and the experimental protocol was approved by the Animal Care Committee of Hebei Medical University (Shijiazhuang, China). A total of 24 New Zealand rabbits, with equal numbers of males and females, 2.2±0.3 kg in weight, were obtained from the Beijing Fuhao Experimental Animal Centre (Grade II, Certificated SCXK JING2010-0010; Beijing, China) and were housed at the Animal Care Centre of the Key Laboratory of Collateral Disease of Hebei Province (Shijiazhuang, China). The rabbits were randomly allocated into two groups of 12 animals each after administration with an adaptive feed (Hebei Province Experimental Animal Centre, Shijiazhuang, China) for 2 weeks. The rabbits in the negative control group (n=12) were fed a normal diet for 4 weeks. The hypercholesterolemic diet group (HC; n=12) were fed a high-cholesterol diet (normal diet supplemented with 1% cholesterol, 7.5% yolk powder and 5% lard oil) for 4 weeks. All animals received access to drinking water *ad libitum* and were on a 12 h light/dark circadian rhythm.

### Specimen collection and processing

At the completion of the experiment, blood samples were obtained from the marginal ear vein following overnight fasting and all animals were sacrificed with an overdose of sodium pentobarbital (100 mg/kg). Subsequently, two rabbits were randomly selected from each group for whole aortic Oil-red O staining (Nanjing Jiancheng Bioengineering Institute, Nanjing, China). The remaining abdominal aortas from 20 animals were removed quickly and cut into two segments. The first segment, obtained from the proximal end ~1 cm down from the diaphragm, was fixed in 4% paraformaldehyde in 0.01 M phosphate buffer (pH 7.4) under physiological pressure for subsequent hypoxia-inducible factor (HIF)-1α and cluster of differentiation (CD)34 immunohistochemical staining. The remaining segment was immediately snap-frozen in liquid nitrogen for molecular assessment.

### Biochemical assay

Serum was obtained via centrifugation at 1,930 × g for 10 min. Serum total cholesterol (TC), triglycerides (TG), high-density lipoprotein cholesterol (HDL-C) and LDL-cholesterol (LDL-C) levels were determined using an automatic biochemical analyser (Hitachi 7080; Hitachi Co., Tokyo, Japan). The serum nitric oxide (NO) level was examined using a nitrate reductase method, employing an NO detection kit (Nanjing Jiancheng Bioengineering Institute, Nanjing, China), which was used according to the manufacturer's instructions. Simultaneously, serum endothelin-1 (ET-1) levels were assayed using a sensitive enzyme-linked immunosorbent assay kit (Abcam, Cambridge, UK).

### Histomorphological and immunohistochemical staining

The abdominal aorta segments, following fixation in 4% paraformaldehyde, dehydration and embedding in paraffin, were cut into serial 4-*µ*m thick cross-sections at 3 mm intervals from the proximal to the distal end for hematoxylin and eosin (H&E) staining and specific immunohistochemical staining. A streptavidin-peroxidase immunohistochemistry kit was obtained and experiments were performed according to the manufacturer's instructions (Zhongshan Golden Bridge Biotechnology Co., Ltd., Beijing, China). Briefly, endogenous peroxidase activity was inhibited by incubation with 3% H_2_O_2_. Sections were blocked with 5% goat serum in phosphate-buffered saline (PBS) and incubated overnight at 4°C with the primary antibodies. Following a PBS wash, the sections were incubated with the corresponding secondary antibody at 37°C for 30 min. Immunohistochemical staining was visualized by use of a 3,3′-diaminobenzidine kit (Zhongshan Golden Bridge Biotechnology Co., Ltd.) according to the manufacturer's instructions. The sections were then counterstained with the nuclear stain, hematoxylin (blue). The primary antibodies included an anti-HIF-1-α (H1α67) antibody (1:50; cat. no. GR1125311; Abcam) and a CD34 antibody (1:300; cat. no. orb129550; Biorbyt, Cambridge, UK).

### Histological analysis

Slides were scanned using a Leica DM6000B microscope (Leica, Wetzlar, Germany). Manual microvessel counting was performed by a single professional observer, in a blinded manner. Each microvessel was defined as a single lumen surrounded by a layer of endothelial cells indicated by immunostaining with the anti-CD34 antibody. Based on the relevant literature (15), microvessels (Q) and the reference areas (A) of the section through the vascular wall were counted in all sections (magnification, ×200). The ratio of the two figures was considered to be the microvessel density value (Q_A_) and expressed as microvessels/mm^2^ of the vascular wall sectional area, as calculated using the following equation: Q_A_ = Q/A.

### Western blotting for HIF-1α, nuclear factor (NF)-κB, tumor necrosis factor (TNF)-α, interleukin (IL)-6, Nuclear factor (erythroid-derived 2)-like 2 (Nrf2), γ-glutamylcysteine synthetase (γ-GCS) and heme oxygenase (HO)-1

The arteries of four rabbits from each group were snap-frozen in liquid nitrogen and subsequently homogenized in lysis buffer (containing 50 mmol/l Tris-HCl, pH 7.5; 150 mmol/l NaCl, 1 mmol/l Na_3_VO_4_, 1 mmol/l phenylmethanesulfonylfluoride, 1 mmol/l dichlorodiphenyltrichloroethane, 1% NP-40 and 10% glycerol) by use of a tissue homogenizer. The lysate was analysed for protein content using the Bradford assay (Bio-Rad Laboratories, Inc., Hercules, CA, USA). Equal quantities of protein were resolved by 10% SDS-PAGE and combined with the following primary antibodies: HIF-1α (mouse monoclonal antibody, 1:1,000, Abcam); NF-κB (goat polyclonal antibody, 1:1,000, Santa Cruz Biotechnology, Inc., Santa Cruz, CA, USA); TNF-α (monoclonal antibody, 1:1,000, Santa Cruz Biotechnology, Inc.); IL-6 (goat polyclonal antibody, 1:1,000, Abcam); Nrf2 (goat polyclonal antibody, 1:1,000, Abcam); γ-GCS (goat polyclonal antibody, 1:1,000, Santa Cruz Biotechnology, Inc.); HO-1 (rabbit polyclonal antibody, 1:1,000, Abcam) and β-actin (mouse monoclonal antibody, 1:1,000, Abcam, Cambridge, MA, USA), at 4°C with slow oscillation overnight. Subsequently, the membranes were probed with a biotin-streptavidin horseradish peroxidase detection system (cat. no. SP-9001; Zhongshan Co., Beijing, China) and a DAB detection kit (cat. no. SP-9000-D; Zhongshan Co.) and developed with chemiluminescence. Densitometry was performed by use of a Labworks system (UVP Products, Upland, CA, USA). β-actin was used as an internal control. The relative expression values of the target gene were normalised via the greyscale ratio of the target gene to β-actin.

### Reverse transcription-quantitative polymerase chain reaction (RT-qPCR)

The tissue samples were frozen with liquid nitrogen. Total RNA was extracted using TRIzol reagent (Invitrogen Life Technologies, Carlsbad, CA, USA). Total RNA was quantified using a 756 ultraviolet spectrophotometer (Thermo Fisher Scientific Inc., Waltham, MA, USA) and reverse transcribed using the M-MLV Reverse Transcriptase system (Promega Corporation, Madison, WI, USA) with random primers. The mRNA expression of HIF-1α, NF-κB, TNF-α, IL-6, Nrf2, γ-GCS and HO-1 in the vessel walls were examined via RT-qPCR using the ABI 7300 Real-Time PCR system (Applied Biosystems, Foster City, CA, USA) according to the manufacturer's instructions. The primer sequences are listed in Table I. Quantitative values were obtained from the threshold cycle value (Ct). An internal control, GAPDH was used to calculate the relative quantitative values of the target genes of each sample.

### Statistical analysis

All data are expressed as the mean ± standard deviation. A two-tailed Student's t-test was used to compare individual groups. All data were analysed using SPSS 18.0 (SPSS Inc., Chicago, IL, USA). P<0.05 was considered to indicate a statistically significant difference.

## Results

### Serum lipid, NO and ET-1 parameters

As shown in Table II, compared with the normal diet group, serum TC, TG, HDL-C, LDL-C and ET-1 levels all increased in the high-cholesterol diet group, while the serum NO level decreased significantly (P<0.01).

### Histomorphology and immunohistochemistry

**Figure 1:**
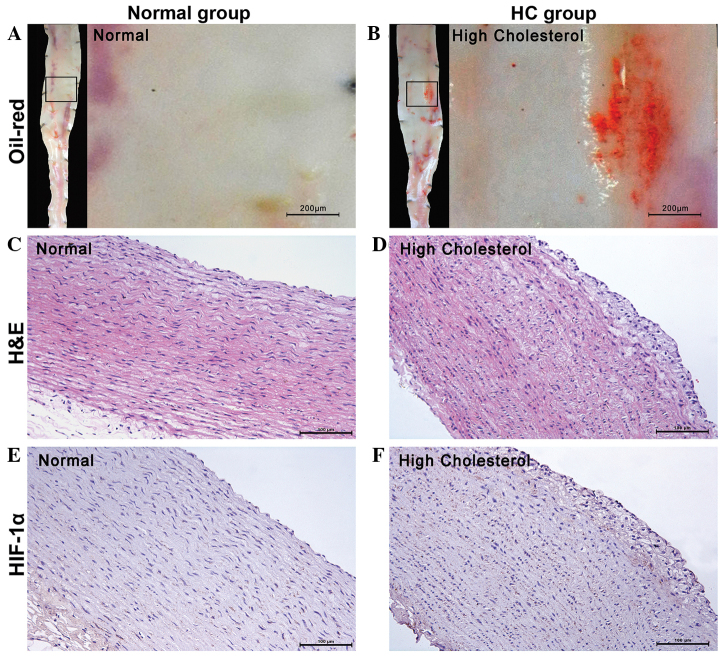
Staining of the abdominal aorta. Whole aortic Oil-red O staining revealing the endothelial surface of the aorta as smooth and white. (A) Normal diet group and (B) HC group with fatty streaks dyed red, (stereoscopic microscope; magnification, ×40; scale bars, 200 *µ*m). Colour photomicrographs demonstrating representative H&E staining and immunostaining of cross sections from the abdominal aorta with an anti-HIF-1α antibody (light microscope; magnification, ×200). (C) Intima is smooth in the normal diet group. (D) Foam cell formation in the HC group. Expression of anti-HIF-1α antibody in (F) the HC group was similar to that in (E) the normal diet group. No marked brown positive expression was observed in either group (scale bars, 100 *µ*m). HC, high-cholesterol diet group; HIF, hypoxia-inducible factor; H&E, hematoxylin and eosin.

At the completion of the experiment, the whole aortic Oil-red O staining revealed the endothelial surface of the aorta was smooth and white in the normal diet group (Fig. 1A), however fatty streaks formed in the HC group (Fig. 1B). In addition, H&E staining demonstrated that the intima is smooth in the normal diet group (Fig. 1C), however foam cell formation in the intima of the rabbit abdominal aorta in the HC group (Fig. 1D). Immunohistochemical staining revealed that the expression of HIF-1α, as indicated by the anti-HIF-1α antibody did not indicate positive expression in either group (Fig. 1E and F).

### Microvessel density in abdominal aortic wall

**Figure 2:**
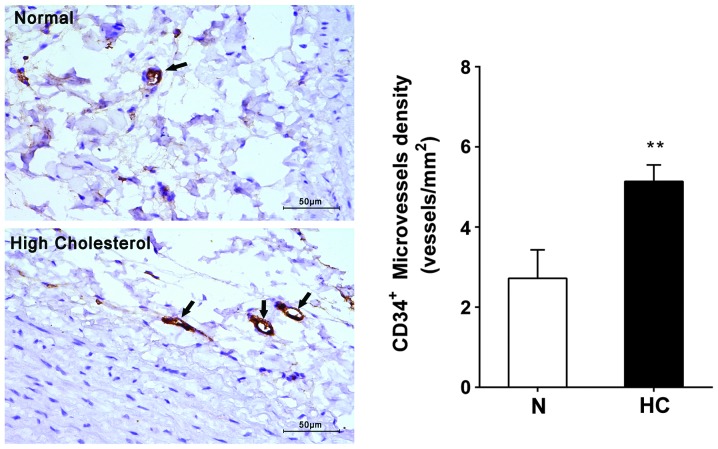
Perivascular CD34^+^ microvessel density is significantly increased in the HC group (^**^P<0.01, right panel, normal diet group: n=10, HC: n=10). Left panel: Representative micrographs (magnification, ×400) of perivascular tissue of normal rabbits (upper) and HC rabbits (lower) stained with an antibody against CD34. The CD34^+^ microvessels are indicated by black arrows (scale bar, 50 *µ*m). HC, high-cholesterol diet group; CD, cluster of differentiation; N, normal diet group.

The immunohistochemical staining revealed that microvessels in the adventitia of the abdominal aortic wall were immunoreactive to the CD34 antibody. The microvessel density of the HC group was 5.14±0.41 vessels/mm^2^, significantly higher than that of normal diet group, at 2.72±0.71 vessels/mm^2^ (P<0.01; Fig. 2).

### Comparison of HIF-1α, NF-κB, TNF-α, IL-6, Nrf2, γ-GCS and HO-1 protein levels

**Figure 3:**
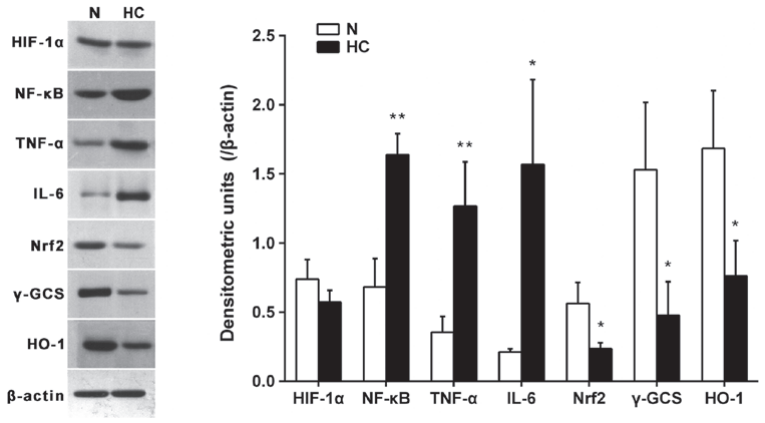
Representative immunoblots of rabbit abdominal aorta tissue homogenates of the two groups for HIF-1α, NF-κB, TNF-α, IL-6, Nrf2, γ-GCS and HO-1 protein expression (left panel). HIF-1α protein level was similar in the N diet group and HC diet group. The expression of NF-κB, TNF-α and IL-6 increased in the HC group. The expression of Nrf2, γ-GCS and HO-1 declined in the HC group. The bar graph illustrates expression levels as a densitometric ratio to β-actin averaged for four samples per group; values are expressed as the mean ± standard deviation, ^*^P<0.05 and ^**^P<0.01 compared with the normal diet group. HC, high-cholesterol diet group; N, normal diet group. HIF, hypoxia-inducible factor; NF, nuclear factor; TNF, tumor necrosis factor; IL, interleukin; Nrf2, nuclear factor (erythroid-derived 2)-like 2; GCS, glutamylcysteine synthetase; HO, heme oxygenase.

The relative protein levels of HIF-1α in the normal diet group and in the HC group were 0.74±0.14 and 0.57±0.09, respectively and no significant difference was identified between the two groups (P>0.05). The relative protein expression levels of the inflammation-associated cytokines, NF-κB, TNF-α and IL-6 in the HC group (1.64±0.15, 1.27±0.32 and 1.57±0.61) were significantly higher than in the normal diet group (0.68±0.20, 0.36±0.11 and 0.21±0.02; P<0.05 or P<0.01), while the relative protein expression levels of the antioxidant-associated factors Nrf2, γ-GCS and HO-1 of the HC group (0.24±0.04, 0.48±0.24 and 0.76±0.26) were significantly lower than that of the normal diet group (0.56±0.15, 1.53±0.49 and 1.68±0.42; P<0.05; Fig. 3).

### mRNA expression of HIF-1α, NF-κB, TNF-α, IL-6, Nrf2, γ-GCS and HO-1

**Figure 4:**
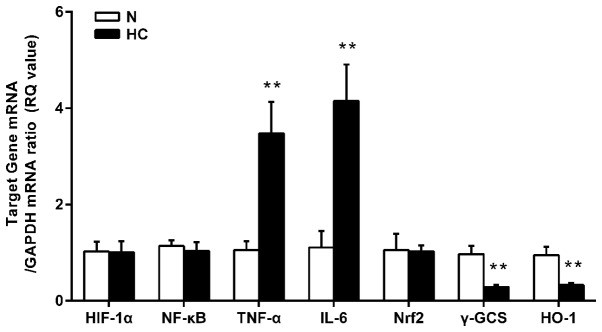
mRNA expression levels of HIF-1α, NF-κB, TNF-α, IL-6, Nrf2, γ-GCS and HO-1 in rabbit abdominal aortas indicated by reverse transcription-quantitative polymerase chain reaction. The expression of HIF-1α mRNA, NF-κB mRNA and Nrf2 mRNA was not significantly different between the N diet group and the HC diet group. The expression of TNF-α mRNA and IL-6 mRNA were higher while the expression of γ-GCS and HO-1 mRNA were lower in the HC group. All data have been standardized against GAPDH mRNA. The RQ values of the target genes are expressed as the mean ± standard deviation, ^*^P<0.05 and ^**^P<0.01, compared with the normal diet group. RQ, relative quantitative; HC, high-cholesterol diet group; N, normal diet group. HIF, hypoxia-inducible factor; NF, nuclear factor; TNF, tumor necrosis factor; IL, interleukin; Nrf2, nuclear factor (erythroid-derived 2)-like 2; GCS, glutamylcysteine synthetase; HO, heme oxygenase.

No significant differences were observed in the mRNA expression levels of HIF-1α, NF-κB and Nrf2 between the normal diet group and the HC group (P>0.05). These findings were not concordant with the results of the western blotting described above. The mRNA expression levels of TNF-α and IL-6 were higher in the HC group than in the normal diet group (P<0.01), whereas the mRNA expression levels of γ-GCS and HO-1 were lower in the HC group than in the normal diet group (P<0.01; Fig. 4).

## Discussion

The present study was designed to address the role of inflammation, oxidative stress and hypoxia on the micro-angiogenesis of early developmental stages of atherosclerosis in rabbits. The initiation lesions of atherogenesis in the rabbit abdominal aorta were formed after 4 weeks administration of a high-cholesterol diet and closely resemble those occurring in humans, including a reduction in serum NO levels, an increase in serum ET-1 levels and in particular, the formation of fatty streaks. The results of the present study demonstrated that micro-angiogenesis of the arterial wall appeared in the initial stage of atherosclerosis. During this time, neither the protein level nor the mRNA level of HIF-1α in the abdominal artery was identified to change significantly. The protein expression of the inflammation-associated factors NF-κB, TNF-α and IL-6 increased and simultaneously, the protein expression of the antioxidant-associated factors Nrf2, γ-GCS and HO-1 were significantly reduced. No significant differences were observed in the mRNA expression of NF-κB and Nrf2 between the two groups. This may be due to the two genes changing during the modification process, which in turn led to changes in the degradation levels. These findings indicated that inflammation and oxidative stress are important in the micro-angiogenesis associated with the initial phase of atherosclerosis, but hypoxia was not the main pro-angiogenic factor during this time period.

Several studies have indicated a potential role for adventitial VV angiogenesis in atherogenesis (4,16,17), however, the studies did not address the mechanism of angiogenesis in the initial phase of atherosclerosis. In addition, with respect to early atherosclerosis induced by a high-cholesterol diet, various theories exist on the contribution of arterial wall hypoxia. A study indicated that hypoxia of the arteries exists in early atherosclerosis (18), while another study demonstrated that hypercholesterolemia does not alter the delivery of oxygen to the arterial wall prior to the formation of atherosclerotic lesions (14). No final conclusion has been reached on this matter at present. The early atherosclerotic lesions induced by a high-cholesterol diet are consistent with the pathogenic characteristics of atherosclerosis in humans, which often follow hyperlipidemia in normal conditions. A number of the previous studies of atherosclerosis were based on an animal model of hyperlipidemia (18,19,20). The present data revealed that levels of inflammatory markers increased and antioxidant levels declined during the angiogenesis of early atherosclerotic lesions. These data suggest that hyperlipidemia possibly activates inflammatory signalling pathways and suppresses the antioxidant signalling pathways, which subsequently promotes angiogenesis.

It has been well-established that HIF-1α is an important marker of hypoxia and HIF-1α promotes angiogenesis by upregulating the expression of vascular endothelial growth factor (VEGF). For example, following the occurrence of ischemic cardiovascular or cerebrovascular disease, the expression of VEGF increases due to local hypoxia, which then promotes angiogenesis, which improves the local blood and oxygen supply. However, the mechanism of angiogenesis may differ from this in early atherosclerotic lesions induced by a high-cholesterol diet. Under normal conditions, the number of microvessels in the arterial wall is relatively balanced. Angiogenesis is beneficial when the arterial wall lacks oxygen; however, if there is no hypoxia in the arterial wall, then this type of angiogenesis, due to the structure and function of incomplete angiogenesis, may increase invasion by inflammatory substances and oxidative products. Thus, unnecessary angiogenesis may increase lipid accumulation in the area, which increases the risk of an atherosclerotic lesion developing to an advanced stage (4,13).

An inflammatory reaction is present at all stages of atherosclerosis, from early endothelial activation to the ultimate plaque rupture. NF-κB is one of the major regulatory factors of inflammation and is considered to be a pro-atherosclerotic factor. It is involved in atherosclerosis via the regulation of a number of the pro-inflammatory genes associated with atherosclerosis. Previous studies have observed that hyperlipidemia is able to induce the activation of NF-κB and may be associated with a decrease in NO bioavailability in the initial stages of atherosclerosis in the pig coronary vasculature (19). The inflammatory mediator TNF-α is able to promote the expression of cell adhesion molecules, which rely on the NF-κB-induced expression of Jagged-1 by endothelial cells to promote angiogenesis (21) and induce angiogenesis through regulating the macrophages (22). IL-6 has a pivotal role in the inflammatory reaction and is an important cytokine involved in various regulatory functions in the body. TNF-α and IL-6 activation is closely associated with NF-κB activation. In addition, previous studies have demonstrated that ET-1 is also a pro-angiogenic factor, which is able to induce an angiogenic phenotype in cultured endothelial cells and stimulate neovas-cularisation *in vivo* (23,24). Activated NF-κB decreases the bioavailability of NO and stimulates the expression of ET-1, which subsequently contributes to micro-angiogenesis of the arterial wall (25). The present results demonstrated that hyperlipidaemia is able to increase the rabbit abdominal aortic protein levels of NF-κB, TNF-α and IL-6 and upregulate the expression of TNF-α and IL-6 mRNA suggesting that the inflammatory response has a role in the angiogenic process in the initial phase of atherosclerosis.

Oxidative stress is defined as an imbalance between oxidation and antioxidants, which is induced by the overproduction of oxidative products or an insufficient production of antioxidants. This imbalance leads to damage of important biomolecules and organs, and may potentially affect whole organisms (26). Reactive oxygen species (ROS) contribute to lipid peroxidation, which is one of the major causes of the formation of atherosclerotic plaques (27). In numerous pathophysiological conditions, intracellular and extracellular ROS are involved in the process of angiogenesis (28). Nrf2 is an important endogenous antioxidant transcriptional regulatory factor. It is able to regulate a variety of antioxidants, including phase II detoxifying enzyme and it affects gene expression throughout the body through the induction of antioxidant response elements (29), which have a central role in the regulation of the oxidative stress response. The γ-GCS and HO-1 genes are the downstream targets of Nrf2 and the two are regulated by Nrf2, which is regarded as one of the most important intracellular antioxidant mechanisms (30). Data from the present study revealed that hyperlipidaemia significantly decreased the protein levels of Nrf2, γ-GCS and HO-1 and downregulated the expression of γ-GCS and HO-1 mRNA, which suggests that angiogenesis is possibly associated with this mechanism in the initial stages of atherosclerosis. The decrease in the expression of antioxidant factors may activate the ROS system, which in turn promotes angiogenesis.

Furthermore, inflammation and oxidative stress may exhibit cross-talk in terms of promoting angiogenesis. Inflammation induces lipid peroxidation and lipid peroxidation in turn gives rise to further inflammation and together, these factors activate NF-κB target gene expression and trigger the first steps in the development of an atherosclerotic lesion. Activation of Nrf2 reduces endothelial cell activation at atherosusceptible sites (31). In addition, the endothelial cell apoptosis induced by oxidative stress is also associated with NF-κB activation (32). Notably, previous studies have revealed that ROS are also important in angiogenesis. ROS trigger lipid oxidation to generate 2-(ω-carboxyethyl) pyrrole (CEP), an end product of lipid oxidation, which in turn induces angiogenesis through activating CEP-Toll-like receptor (TLR)2/1 signalling. This is a novel VEGF-independent signalling pathway, which promotes angiogenesis (33). Another recent finding has implicated an alternative mediator of oxidative stress-induced pathological angiogenesis, ataxia telangiectasia mutated (ATM) kinase. ATM activation by an increase in ROS levels was revealed to promote pathological neoangiogenesis. Notably, ATM appears to be uniquely involved in pathological angiogenesis, but not in physiological vascularization (34). These findings demonstrate how oxidation and inflammation are able to orchestrate angiogenesis. Together, ROS mediate various signalling pathways that underlie vascular inflammation in atherogenesis, from the initiation of fatty streak development, through lesion progression to the ultimate plaque rupture.

The limitations of the present study in terms of design and methods must be acknowledged. For example, the levels of HIF-1α, inflammatory factors and antioxidant-associated factors of the rabbit arterial wall were only observed after 4 weeks of high-cholesterol feeding therefore, these parameters were not continuously monitored. With further development of an atherosclerotic lesion, including arterial wall thickening and increasing oxygen consumption, hypoxic conditions may appear. In addition, it was not examined whether there were the same alterations in gene and protein expression in the coronary artery, carotid artery or other arteries of the assessed rabbits, or in other animal models. There may be differences between species or vascular beds due to different signalling pathways, which in turn may involve different angiogenic mechanisms during the development and progression of atherosclerosis. Therefore, the findings of the present study should be assessed in different vascular beds and alternative animal models. Our future investigations aim to address these points.

The present data indicated that hypoxia is not evident in the arterial wall during the initial stages of the formation of hyperlipidemia-induced atherosclerotic lesions. The increase in the level of inflammatory markers and decrease in antioxidant function are likely to be the major factors involved in angiogenesis within the arterial wall at this stage. It is necessary to examine the basic pathological mechanism of atherosclerosis to prevent cardiovascular and cerebrovascular diseases and to produce more effective intervention strategies.

## Figures and Tables

**Table I:** Primer sequences used in reverse transcription-quantitative polymerase chain reaction.

Gene	Primer sequence (5′-3′)	Product (bp)
HIF-1α		
Sense	CCTGCCTCTGAATCTCCAA	147
Antisense	AGAAGGACTTGCTGGCTGA	
NF-κB		
Sense	GGCTTGCGTTCAGATGTTG	73
Antisense	AACCGTCTTGTTGCTCTTGA	
TNF-α		
Sense	AGTAGCAAACCCGCAAGTG	148
Antisense	GCTGAAGAGAACCTGGGAGTA	
IL-6		
Sense	AACCAGTGGCTGAAGACGA	62
Antisense	TCCAGGAAGTTTGTGAGGC	
Nrf2		
Sense	TTCTTTCGGCAGCATCCT	62
Antisense	TGGCATTTGAGTTCACGC	
γ-GCS		
Sense	ACCTCCTCCAAACTCCGAT	102
Antisense	AGCACCACAAACACCACATAG	
HO-1		
Sense	GCCACCAAGTTCAAGCAG	88
Antisense	TAGCCTCTTCCACCACCCT	
GAPDH		
Sense	AGAGATTGTGCGGGATGTC	92
Antisense	CCAGTGAGGAAGATGCTGCT	

HIF, hypoxia-inducible factor; NF, nuclear factor; TNF, tumor necrosis factor; IL, interleukin; Nrf2, nuclear factor (erythroid-derived 2)-like 2; GCS, glutamylcysteine synthetase; HO, heme oxygenase.

**Table II:** Comparison of the levels of serum lipids, NO and ET-1 in the experimental groups.

Group	TC (mmol/l)	TG (mmol/l)	HDL-C (mmol/l)	LDL-C (mmol/l)	NO (*µ*mol/l)	ET-1 (pg/ml)
N	3.28±0.93	0.76±0.17	0.68±0.17	1.80±0.59	17.22±2.66	46.99±5.13
HC	39.96±6.13^a^	3.99±0.78^a^	3.77±0.96^a^	26.76±4.67^a^	13.06±1.85^a^	59.89±5.51^a^

Data are expressed as the mean ± standard deviation (^a^P<0.01, compared with normal diet group; n=12). N, normal diet group; HC, high-cholesterol diet group; TC, total cholesterol; TG, triglycerides; HDL-C, high-density lipoprotein cholesterol; LDL-C, low-density lipoprotein cholesterol; NO, nitric oxide; ET, endothelin.
